# Intracellular development and impact of a marine eukaryotic parasite on its zombified microalgal host

**DOI:** 10.1038/s41396-022-01274-z

**Published:** 2022-07-08

**Authors:** Johan Decelle, Ehsan Kayal, Estelle Bigeard, Benoit Gallet, Jeremy Bougoure, Peta Clode, Nicole Schieber, Rachel Templin, Elisabeth Hehenberger, Gerard Prensier, Fabien Chevalier, Yannick Schwab, Laure Guillou

**Affiliations:** 1 Laboratoire Physiologie Cellulaire et Végétale, Univ. Grenoble Alpes, CNRS, CEA, INRAE, IRIG-DBSCI-LPCV, 38000 Grenoble, France; 2 Sorbonne Université, CNRS, UMR7144 Adaptation et Diversité en Milieu Marin, Ecology of Marine Plankton (ECOMAP), Station Biologique de Roscoff, 29680 Roscoff, France; 3 Laboratoire de Biologie des Organismes et Ecosystèmes Aquatiques (BOREA)—Université de Caen Normandie, MNHN, SU, UA, CNRS UMR 8067, IRD 207, 14000 Caen, France; 4 Institut de Biologie Structurale (IBS), University Grenoble Alpes, CEA, CNRS, 38044 Grenoble, France; 5 The Centre for Microscopy Characterisation and Analysis, The University of Western Australia, Perth, WA 6009 Australia; 6 UWA School of Biological Sciences, The University of Western Australia, Perth, WA 6009 Australia; 7 Cell Biology and Biophysics Unit, European Molecular Biology Laboratory (EMBL), 69117 Heidelberg, Germany; 8 Institute of Parasitology, Biology Centre, Czech Academy of Sciences, České Budějovice, Czech Republic; 9 Université François-Rabelais, Laboratoire Biologie cellulaire et Microscopie électronique, 37032 Tours, France; 10 Present Address: Ramaciotti Centre for Cryo Electron Microscopy, Monash University, Clayton, 3800 VIC Australia

**Keywords:** Ecology, Structural biology, Transcriptomics

## Abstract

Parasites are widespread and diverse in oceanic plankton and many of them infect single-celled algae for survival. How these parasites develop and scavenge energy within the host and how the cellular organization and metabolism of the host is altered remain open questions. Combining quantitative structural and chemical imaging with time-resolved transcriptomics, we unveil dramatic morphological and metabolic changes of the marine parasite *Amoebophrya* (Syndiniales) during intracellular infection, particularly following engulfment and digestion of nutrient-rich host chromosomes. Changes include a sequential acristate and cristate mitochondrion with a 200-fold increase in volume, a 13-fold increase in nucleus volume, development of Golgi apparatus and a metabolic switch from glycolysis (within the host) to TCA (free-living dinospore). Similar changes are seen in apicomplexan parasites, thus underlining convergent traits driven by metabolic constraints and the infection cycle. In the algal host, energy-producing organelles (plastid, mitochondria) remain relatively intact during most of the infection. We also observed that sugar reserves diminish while lipid droplets increase. Rapid infection of the host nucleus could be a “zombifying” strategy, allowing the parasite to digest nutrient-rich chromosomes and escape cytoplasmic defense, whilst benefiting from maintained carbon-energy production of the host cell.

## Introduction

In aquatic and terrestrial ecosystems, some organisms have developed adaptations to benefit and exploit the metabolism of other organisms through many forms of symbiosis, ranging from commensalism to mutualistic and parasitic interactions. Parasites are recognized as important elements in the function and resilience of ecosystems and for the evolution of organisms. While research has largely focused on human and domestic animal parasites, there is a newfound awareness of the relevance of planktonic parasites, particularly in marine ecosystems [1]. In the past decade, an increasing diversity of eukaryotic parasites in the ocean, such as Syndiniales, Perkinsozoa and Chytridiomycota, have been characterized using a combination of DNA sequencing and microscopy [2–5]. These parasites are widely distributed in the oligotrophic open ocean and coastal waters [6–8]. Several of these parasites infect planktonic microalgae (single-celled photosynthetic eukaryotes), possibly taking advantage of the highly valuable carbon resources produced by the host photosynthetic machinery. With this, parasites can affect algal population dynamics [9], which is of high ecological and economic importance, for example, when high mortality rates cause a decline of bloom-forming toxic microalgae in coastal areas (Chambouvet et al., 2008; Montagnes et al., 2008).

The most frequent and diversified marine parasites are Syndiniales (a deep branching lineage of dinoflagellates), which have a relatively narrow host spectrum. Throughout their evolution, Syndiniales have likely lost their plastids (i.e. in all species described so far including *Amoebophrya* and *Hematodinium*), and there is no evidence of a vestigial organelle in their cytoplasm [10–12], like the apicoplast found in apicomplexans [13, 14]. While many parasites, such as *Parvilucifera* and *Dinomyces*, kill their host before digesting them [15], most Syndiniales keep their host alive throughout most of the infection period (also called biotrophic parasitoids) [3, 16]. For instance, the obligate and specialist parasite *Amoebophrya* spp. infects dinoflagellates [3, 17], which remain photosynthetically active during most of the internal development of the parasite [18]. This infection strategy very likely allows the parasite to efficiently exploit the carbon metabolism of the host (e.g. photosynthetic products), thereby enhancing growth and replication. While host organelles are physiologically active during infection of *Amoebophrya*, it is not clear how host energy production and carbon storage are impacted by the presence of the parasite. At the end of the infection, the parasite releases numerous motile, flagellated infectious zoospores (called dinospores), which do not divide and therefore have a short timeframe (3–15 days), in which to find a new host [9, 19]. Syndiniales are therefore strongly dependent on the nutrients and metabolites obtained during their intracellular developmental stages *in hospite* to grow and meet their energy demand while searching for a new host.

To date, little mechanistic knowledge is available on the intracellular development of the parasite during infection and its impact on the overall metabolism of its algal host. Fundamental aspects of the parasitic infection of Syndiniales are still unclear, especially regarding the underlying subcellular mechanisms taking place inside the host cell. To fill this knowledge gap, we used three-dimensional (3D) electron microscopy combined with transcriptomics to understand the infection strategy of the parasite *Amoebophrya* sp. (strain A120) within its microalgal host (*Scrippsiella acuminata*, Dinophyceae). We investigated the concomitant structural development of the parasite and its impact on the host at the subcellular level. Our approach revealed major morphological and metabolic shifts during intracellular development of the parasite. By contrast, the bioenergetic machinery of the host is only slightly impacted, suggesting that carbon production by the host (starch and lipids) potentially fuels the metabolism of the parasite. Overall, this study provides unprecedented mechanistic insights into a widespread and ecologically important parasite infecting marine phytoplankton. Given that several of these strategies are common to apicomplexan parasites (e.g. *Plasmodium falciparum* and *Toxoplasma gondii*), our study also offers new insights into the evolution of parasitism in Alveolata and in eukaryotes more generally.

## Methods

### Culture conditions

The dinoflagellate *Scrippsiella acuminata* ST147 (RCC 1627) was maintained in F2 medium (enriched with 5% of soil v/v) in these following culture conditions: 20 °C, 80–100 µmol photons m^−2^s^−1^, L:D cycle of 12:12 h. (More information on the culture provided here: dx.doi.org/10.17504/protocols.io.vrye57w). The parasite *Amoebophrya ceratii* A120 (RCC 4398) (Syndiniales, Amoebophryidae, equivalent to Marine ALVeolates Group II, or MALV-II) was maintained by inoculating frequently (every 3–4 days) fresh cultures of *Scrippsiella acuminata* (3–4 days old). For the infection experiment, dinospores (*ex hospite*) of the parasite *Amoebophrya* were obtained by filtering by gravity on a 5 µm mesh size polycarbonate filter and were mixed with a fresh culture of host cells (1 vol dinospore cells for 2 volumes of host cells) for 35 h.

### 2D and 3D Electron microscopy (TEM and FIB-SEM)

#### Sample preparation

The non-infected and infected microalgae *Scrippsiella* were concentrated on a 5 µm mesh size polycarbonate filter. Cells were then collected and cryo-fixed using high-pressure freezing (HPM100, Leica), followed by freeze-substitution (EM ASF2, Leica) as in [20, 21]. For the freeze substitution (FS), a mixture 2% (w/v) osmium tetroxide and 0.5% (w/v) uranyl acetate in dried acetone was used for FIB-SEM (Focused Ion Beam- Scanning Electron Microscopy) with a programed protocol from [20]. For TEM and nanoSIMS, the FS mix contained only 1% of osmium tetroxide. For TEM analysis, ultrathin sections of 60 nm thickness were mounted onto copper grids or slots coated with formvar and carbon. Sections were then stained in 1% uranyl acetate (10 min) and lead citrate (5 min). Micrographs were obtained using a Tecnai G2 Spirit BioTwin microscope (FEI) operating at 120 kV with an Orius SC1000 CCD camera (Gatan).

FIB-SEM acquisition**:** Samples were mounted onto the edge of a SEM stub (Agar Scientific) using silver conductive epoxy (CircuitWorks) with the trimmed surfaces facing up and towards the edge of the stub. Samples were gold sputter coated (Quorum Q150RS; 180 s at 30 mA) and placed into the FIB-SEM for acquisition (Crossbeam 540, Carl Zeiss Microscopy GmbH). Atlas3D software (Fibics Inc. and Carl Zeiss Microscopy GmbH) was used to perform sample preparation and 3D acquisitions. First, a 1 µm platinum protective coat (20–30 µm^2^ depending on ROI) was deposited with a 1.5 nA FIB current. The rough trench was then milled to expose the imaging cross-section with a 15 nA FIB current, followed by a polish at 7 nA. The 3D acquisition milling was done with a 1.5 nA FIB current. For SEM imaging, the beam was operated at 1.5 kV/700 pA in analytic mode using an EsB detector (1.1 kV collector voltage) at a dwell time of 8 µs with no line averaging. For each slice, a thickness of 8 or 10 nm was removed, and the SEM images were recorded with a pixel size of 8 or 10 nm, providing an isotropic voxel size of 512 nm^3^ or 1000 nm^3^. Raw electron microscopy data are deposited in EMPIAR, accession code EMPIAR- 47484134.

#### 3D reconstruction and volume quantification

From the stack of images, regions of interest were cropped using the open software Fiji (https://imagej.net/Fiji), followed by image registration (stack alignment), noise reduction, semi-automatic segmentation, 3D reconstruction of cells and morphometric analysis as described previously [22]. Image registration was done by the FIJI plugin “Linear Stack Alignment with SIFT” [23], then fine-tuned by AMST [24]. Aligned image stacks were filtered to remove noise and highlight contours using a Mean filter in Fiji (0.5-pixel radius). Segmentation of organelles (plastids, mitochondrion, nucleus) and other cellular compartments of the parasite and the host cells (starch, lipid) was carried out with 3D Slicer software [25] (www.slicer.org), using a manually-curated, semi-automatic pixel clustering mode (5 to 10 slices are segmented simultaneously in z). We assigned colors to segmented regions using paint tools and adjusted the threshold range for image intensity values. Morphometric analyses were performed with the 3D slicer module “segmentStatistics” on the different segments (segmented organelles) and converted to µm^3^ considering the voxel size of 512 or 1000 nm^3^ (Table S1). In total, we analyzed three non-infected host cells, six infected host cells, and 13 parasites.

### NanoSIMS measurements

Semi-thin sections (200–300 nm) on silicon wafers were coated with 20-nm gold-palladium and analyzed with a nanoSIMS 50 L (Cameca, Gennevilliers, France) at the Center for Microscopy, Characterisation and Analysis (The University of Western Australia). A 16-keV Cs+ primary ion beam of ~0.75 pA (D1 = 3) focused to approximately 70 nm was rastered over the 25 µm^2^ sample area (256×256 pixel), with a dwell time of 60 ms/pixel. Before analysis, each area was pre-implanted with a ~3 × 10^16 ^ions per cm^2^. Detectors (electron multipliers) were positioned to simultaneously measure negative secondary ions (^12^C^14^N, ^31^P^16^O_2_, ^34^S, ^12^C_2_). Mass resolving power was optimised using Entrance slit 3 (20 µm), aperture slit 2 (200 µm) and energy slit 1(~10% yield reduction) and calculated as being ~9000 (^12^C^14^N detector) according to Cameca’s MRP definition – sufficient to resolve all ion species of interest. Based on the secondary ion ^12^C^14^N count map, two regions of interest (ROI) were defined by manual drawing (parasite cell) and thresholding (host chromosomes) with the look@nanosims software [26]. Ion counts (normalized by scans and pixels number) and ratios (^12^C^14^N/^12^C_2_, ^31^P^16^O_2_/^12^C_2_, ^34^S/^12^C_2_) were calculated for each ROI (Table S3). Ratio analyses do not provide absolute quantification of nitrogen (N), phosphorous (P) and sulfur (S) concentration but a comparison of the relative content of these elements between ROIs (host chromosomes vs parasite cell). In total, 131 host chromosomes were measured from 27 infected microalgal cells and 22 parasite cells.

### Transcriptomics analyses

#### Curating of enzymes involved in the metabolic pathways and sugar transport of the parasite *Amoebophrya* (Syndiniales)

Reference proteins of interests were downloaded from the UniProtKB (https://www.uniprot.org) and VEuPathDB (https://veupathdb.org/veupathdb/app) databases (last access October 2021). Reference sequences for Sugars Will Eventually be Exported Transporters (SWEET) were obtained from a previous study [27]. These reference sequences were used as BLAST queries to identify homologs in the *Amoebophrya* genome (available here: http://application.sb-roscoff.fr/blast/hapar/download.html) and the identity of positive hits was confirmed by (1) reverse-BLAST to the UniProtKB database (https://www.uniprot.org/blast/; last access October 2021); (2) sequence search in InterPro (http://www.ebi.ac.uk/interpro/; last access October 2021); (3) domain search with Pfam 34.0 (http://pfam.xfam.org/; last access October 2021); (4) phylogenetic analysis as described in [18]. Briefly, homologous sequences were downloaded from public databases, aligned with *Amoebophrya* sequences using mafft v. 7.407 [28], and the alignments were filtered with Gblocks v. 0.91b [29]. Single gene phylogenetic trees were reconstructed for each alignment using RAxML v. 8.2.12 [30] and the tree visually inspected using FigTree v1.4.4 (http://tree.bio.ed.ac.uk/software/figtree/). SWEET-like phylogeny was limited to Myzozoa (Apicomplexa + Dinoflagellata) given the very divergent sequences found in eukaryotes. For each gene, the presence of transmembrane domains was evaluated using the TMHMM Server v. 2.0 (http://www.cbs.dtu.dk/services/TMHMM/). Subcellular location and signal peptides were identified suing TargetP v. 2.0 (https://services.healthtech.dtu.dk/service.php?TargetP-2.0) and SignalP v. 5.0 (https://services.healthtech.dtu.dk/service.php?SignalP-5.0), respectively. For sugar transporters, we assumed positive identification when the number of TMs were similar to those of reference homologs, i.e. 7 for SWEET and 12 for hexose transporters and the phylogenetic position of the *Amoebophrya* gene fell within the *myzozoan* clade (Alveolata excluding ciliates).

##### Gene expression analysis

We used the DESeq2 differential expression analysis tool from the Trinity v2 package [31] to monitor the abundance of the genes of interest in the metatranscriptome (combining the transcriptomes of the host and parasite) produced by previous studies [18, 32] throughout the infection and in dinospores. In short, filtered RNA-seq reads for each replicate (infected cells were sampled in triplicates every 6 h during a 36 h-long infection cycle) were separately mapped with the Bowtie2 aligner module of Trinity, and gene expression matrices were computed using the RSEM method [33]. Not cross-sample normalized transcript per million (TPM) values were calculated for each species, and each time step separately. Only time steps corresponding to an average of 3x coverage of the transcriptome of the parasite were retained for interpretation in this study, namely two replicates at 18 h (A and B) and all replicates for the following time steps (24, 30, 36 and dinospore), based on overall host and parasite remapping values (Table S4). For each gene, the time step where expression was maximum was estimated as the percentage of the average of replicates for each time step divided by the maximum average expression over the whole life cycle. Heatmaps of gene expression values were created using Heatmapper (http://www.heatmapper.ca/).

##### Identification of host transcripts

Host transcripts involved in starch synthesis/degradation as well as type-2 fatty acid and lipid droplet biosynthesis were identified using reference proteins as queries in BLASTP searches against the predicted peptides of the uninfected host. The identity of the recovered host proteins was confirmed by adding them either to existing phylogenies or by reconstructing novel single-protein trees.

## Results and discussion

### Morphological changes of the parasite during intracellular development

**Fig. 1:**
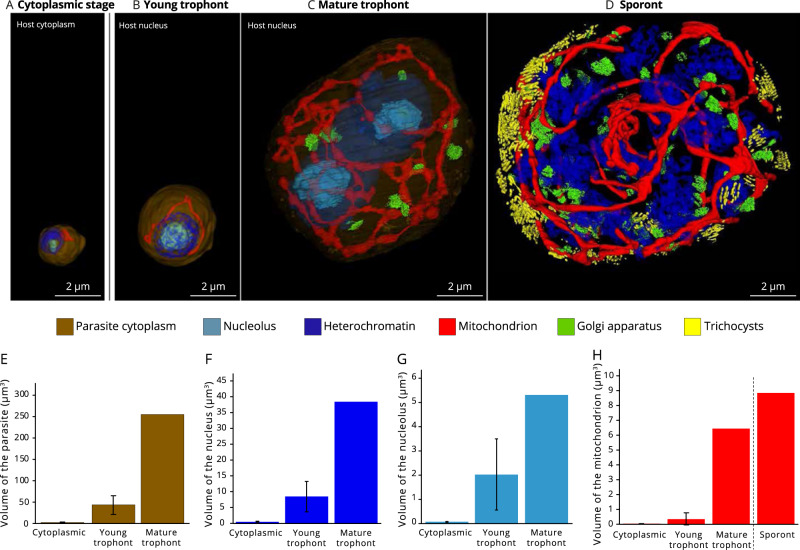
Intracellular development of the marine parasite *Amoebophrya* (Syndiniales) inside its microalgal host (the dinoflagellate *Scrippsiella acuminata*) unveiled by volume electron microscopy (FIB-SEM: Focused-Ion beam Scanning Electron Microscopy). **A** 3D reconstruction of the first infection stage (cytoplasmic parasite) in the host cytoplasm where the parasite displayed a relatively small mitochondrion and condensed chromatin (heterochromatin) at the periphery of the nucleus (Scale bar: 2 µm). **B**, **C** The parasite then invaded the host nucleus where it developed from a young (B) to a mature trophont (C): the volumes of the parasite, its nucleolus and mitochondrion increased. The Golgi apparatus and nucleus division only appear in the mature trophont. (Scale bar: 2 µm). **D** The sporont parasite exhibited multiple nuclei (without visible nucleolus) and Golgi apparatus, and an extended mitochondrion that is dispersed throughout the whole parasite cell volume. Trichocysts were also synthetized at this stage, which are involved in host attachment for new infection. (Scale bar: 2 µm). Brown: parasite volume; light blue: nucleolus; Red: mitochondrion; dark blue: heterochromatin; green: Golgi apparatus; Yellow: trichocysts. **E–H** Volume of the parasite and its organelles (nucleus, nucleolus, mitochondrion) assessed after FIB-SEM-based 3D reconstruction (µm^3^) from four cytoplasmic parasites, seven young trophonts, one mature trophont and one sporont. See also Table S1 for morphometrics data.

Cultures of the photosynthetic dinoflagellate *Scrippsiella acuminata* (here after referred to as “host”) were infected by the Syndiniale parasite *Amoebophrya* sp. (strain A120). In order to understand intracellular development within the host, the parasite and its different organelles were reconstructed in 3D after FIB-SEM (Focused Ion Beam Scanning Electron Microscopy) and their volume assessed across the period of infection. In the life cycle of *Amoebophrya*, four distinct stages have been described: dinospore, trophont, sporont and vermiform [19, 34]. By combining cryo-fixation with high lateral resolution and 3D information, we have further distinguished key developmental steps at the trophont and sporont stages (Fig. 1, Table S1): (1) a transient step hereafter referred to as “cytoplasmic”, whereby the parasite of 2.2 ± 1.0 µm^3^ (*n* = 4) is first located in the host cytosol just after invasion and surrounded by a parasitophorous vacuole; (2) the young round trophont (44.4 ± 22.1 µm^3^, *n* = 8) and (3) the mature amoeboid trophont (up to 266 µm^3^), both in the host nucleus; and (4) the sporont, which occupied most of the host volume. On average, the cytoplasmic stage of the parasite occupied between 0.05 and 0.35% of the host volume, while the young and mature trophonts occupied 2–4% and 19% of the host volume, respectively (Table S1). Multiple trophonts (located in both the host cytoplasm and nucleus) could be observed simultaneously within a single host cell (Fig. S1). During development of these stages, the volume of the parasite cell increased up to 200-fold with dramatic changes in the morphology and volumes of organelles such as the mitochondrion, condensed chromatin, and the development of Golgi apparatus and trichocysts. One of the major morphological changes of the intracellular parasite is seen in the mitochondrion, which developed from a small organelle into a reticulate network throughout the course of the infection (Fig. 1A–D). Compared to the mitochondrion of the cytoplasmic stage of the parasite (0.040 ± 0.005 µm^3^), the volume of this organelle increased by 160 times and 220 times in the mature trophont (6.4 µm^3^) and sporont (8.8 µm^3^), respectively. Similar mitochondrial development is seen in the apicomplexans *Plasmodium falciparum* and *Toxoplasma gondii*, and in the kinetoplastid *Trypanosoma brucei*, where the mitochondrion elongates as a single tube and then forms a branched structure without undergoing fission [35–39]. The mitochondrial network is separated during parasite cytokinesis, suggesting that this significant mitochondrial growth in *Amoebophrya* and other parasites may be a mechanism that ensures a mitochondrion to be distributed to individual dinospores, before sporulation.

The nucleus and its constituents were also modified during development of the parasite. Initially condensed in both dinospores and cytoplasmic parasites, chromatin was gradually decondensed during the trophont stages, supporting the hypothesis that the early cytoplasmic parasite is a transient stage with minimal transcriptional activity (Fig. 1A, B) [19]. In addition, the volume of the nucleus and nucleolus of the trophont increased by about 13 times and 30 times, respectively, compared to the cytoplasmic stage (Fig. 1; Table S1). As ribosome biogenesis is the main function of the nucleolus, we investigated in parallel the expression level of the nuclear ribosomal genes of the parasite in time-resolved transcriptomics data. We found higher expression levels at T24 h and T30 h (Fig. S2), confirming that transcriptional activity and ribosome production concomitantly increase during the trophont stages. The Golgi apparatus first appeared at the mature trophont stage and increased in number within the sporont, suggesting that the cellular machinery for protein and lipid production/maturation activates in these later developmental stages. Karyokinesis was observed in the sporont, visualized by numerous nuclei with peripherally condensed chromatin (lacking a visible nucleolus), but without cytokinesis (Fig. 1D). This pattern resembles replication described in apicomplexans, where daughter cells are formed *de novo* within the cytoplasm without binary fission, along with an elongated mitochondrion [40]. Trichocysts, which are thought to be involved in the attachment of the parasite to a host cell [19] were also synthesized at the sporont stage, presumably in preparation for dinospore formation, release and re-infection of new host cells (Fig. 1D). On average, ~100 dinospores per infected host cell will be produced at the end of the infection [41].

After cellular invasion, the host nucleus appeared to be the main subcellular target, where the parasite settled and rapidly accelerated development and metabolism. The sixfold increase in parasite volume concomitant with growth of the nucleus and mitochondrion, and the formation of the Golgi apparatus between the young and mature trophont stages demonstrates that significant development of the parasite is accomplished inside the host nucleus. Remarkably, nothing is known regarding the trophic strategy of the parasite nor the fate of the host nucleus and its components (e.g. chromosomes).

### Trophic switch to phagotrophy: Degradation and digestion of host chromosomes

**Fig. 2:**
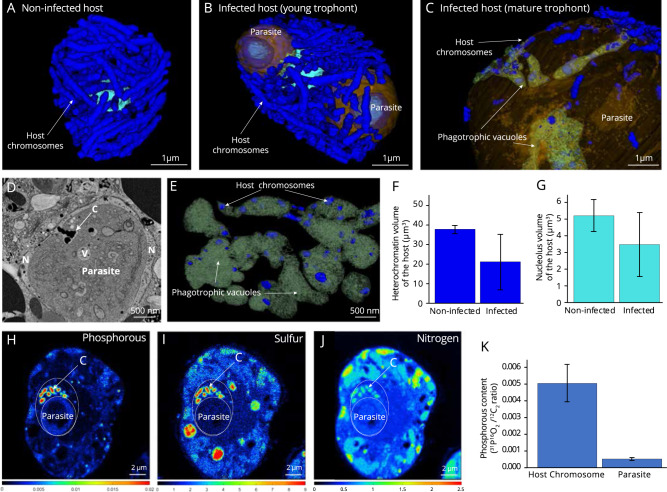
Degradation and digestion of host chromosomes and nucleus by the parasite *Amoebophrya* unveiled by 3D electron microscopy and nanoSIMS. **A** 3D reconstruction of the nucleolus and individual chromosomes of non-infected hosts (about 113–119 per host cell of about 0.33 ± 0.10 µm^3^ each; *n* = 346). **B** Host nucleus, infected by two trophont parasites, displayed smaller chromosomes and nucleolus compared to non-infected hosts. **C–E** At later infection stages, the mature trophont parasite developed multiple phagotrophic vacuoles to engulf and ingest host chromosomes. **D** Electron microscopy micrograph showing the engulfment of an electron-dense host chromosome (C) into the vacuole (V) of a mature trophont parasite within the host nucleus (N). **F**, **G** Volumes of the heterochromatin and nucleolus (in µm^3^) of non-infected and infected host cells assessed after FIB-SEM-based 3D reconstruction. **H–J** NanoSIMS (Nanoscale Secondary Ion Mass Spectrometry) mapping of the elements Phosphorous (H, ^31^P^16^O_2_/^12^C_2_), Sulfur (I, ^34^S/^12^C_2_) and Nitrogen (J, ^12^C^14^N/^12^C_2_), showing that host chromosomes (C) are highly concentrated in these nutrients compared to the nuclear parasite. (Scale bar: 2 µm). **K** Phosphorous (P) content calculated as ^31^P^16^O_2_/^12^C_2_ from nanoSIMS ion count map in the host chromosomes and parasite cell (including nucleus and cytoplasm). P content of the host chromosomes (*n* = 131) were estimated to be about 10 times more important than in the parasite cell (*n* = 22) (See also Table S3). Brown: parasite; light blue: nucleolus; dark blue: heterochromatin. See also Table S1 for morphometrics data.

In non-infected cells, the host nucleus contained ~113–119 individual chromosomes (condensed chromatin) with a volume of 0.33 ± 0.10 µm^3^ each (n = 346 chromosomes), representing a total biovolume of 37 ± 2 µm^3^ within the cell (*n* = 3 cells) (Fig. 2A, Table S1). When a young trophont could be detected in the host nucleus, the volume of individual host chromosomes decreased about 3.5 times (in one infected cell: 0.09 µm^3^ ± 0.04 µm^3^, *n* = 125 chromosomes) (Fig. 2B–F, Table S1). This observed degradation of DNA could explain the steady decline in the number of total host transcripts described previously [18], since less template would be available for transcription. Similarly, a decrease in the volume of the host nucleolus from 5.1 ± 0.9 µm^3^ in non-infected hosts (*n* = 3 cells) down to 2.7–1.9 µm^3^ in infected hosts also suggests lower transcriptional activity that may lead to diminished ribosome production (Fig. 2G, Table S1). Degradation of the host genetic material can potentially be triggered by the parasite. However, we were unable to unambiguously identify genetic signatures related to extracellular chromosome degradation (e.g. nucleases participating in purine and pyrimidine metabolism) in the genome of the *Amoebophrya* parasite. Nevertheless, given that the young trophont parasite is surrounded by an intact and relatively thick membrane with no cytoplasmic invagination, we suggest that import of nutrients and metabolites in the first stages of its intranuclear development could only be through osmotrophy.

By contrast, in the mature trophont, multiple phagotrophic vesicles were observed indicating a switch of the trophic mode from osmotrophy to phagotrophy (Fig. 2C–E). The 3D reconstructions revealed invaginations of the trophont cytoplasmic membrane at several locations creating a tubular network of vacuoles (previously described as a cytopharynx [34]), in which degraded chromosomes of the host (0.03 ± 0.01 µm^3^) were engulfed and digested. In order to further understand this trophic switch, we investigated the expression levels of genes encoding key proteins involved in the formation and acidification of vacuoles during phagocytosis [42–45]: subunits of the vacuolar H^+^-ATPase (V-ATPase) and the GTPase Ras-related protein Rab-11. Both Rab11 and V-ATPase subunits were not expressed in dinospores, but exhibited maximum expression at T24 h and T30 h-T36 h, respectively, corresponding to late intracellular parasite stages (i.e. trophont and sporont) (Fig. S3, Table S2). We hypothesize that Rab11 expression could be related to the formation of phagotrophic vacuoles that eventually fuse with lysosomes, whereas V-ATPase activity generates an acidic local environment for chromosome digestion. Further studies are now required to fully characterize the underlying molecular mechanisms of intranuclear phagotrophy in this parasite.

Subcellular nutrient mapping by NanoSIMS (Nanoscale Secondary Ion Mass Spectrometry) imaging of cellular sections showed that chromosomes of the algal host were rich in phosphorous (P), sulfur (S), and nitrogen (N), representing a concentration hotspot of these nutrients in the host cell (Fig. 2H–K, and S4). For instance, P (^31^P^16^O_2_/^12^C_2_) and S (^34^S/^12^C_2_) content were estimated to be ~9.8 and ~8.5 times higher in host chromosomes (n = 131) than in the parasite cells (*n* = 22), respectively (Table S3). Similarly, N content (^12^C^14^N/^12^C_2_) was ~1.8 times higher in host chromosomes compared to parasite cells. High S content in chromosomes has also been observed in other dinoflagellates [46, 47], and could be explained by chromatin-associated proteins that are rich in cysteine and methionine. Although nutrient transfer cannot be unambiguously demonstrated here, we hypothesize that the parasite gains nutritional benefits from the degradation and digestion of nutrient-rich host chromosomes. DNA could also be a valuable source of carbon for the parasite [48]. Rapid infection of the host nucleus therefore appears to be a key strategy in gaining direct access to major nutritional resources from the host, which are required for parasite growth and replication (e.g. C, N, P), while also escaping cytoplasmic host defense mechanisms. In line with this hypothesis, the substantial increase in the volume of the parasite and its developing organelles (e.g. nucleus, mitochondrion, and the Golgi apparatus) (Fig. 1C) clearly reflects a strategic shift in parasite metabolism and growth during the phagotrophic stage.

During this phagotrophic stage, we also observed significant development of a network of tubules, which were of parasite origin, within the host nucleus. These tubules closely resemble the Intravacuolar Network (IVN) described in the human parasite *Toxoplasma gondii* [49, 50]. During *Amoebophrya* infection, these IVN-like structures often surrounded and concentrated around host chromosomes (Fig. S5). The IVN has been proposed to be involved in nutrient and lipid uptake in *T. gondii* [51, 52] and could play the same role here in this planktonic parasite. Yet, we were not able to identify homologs in the *Amoebophrya* genome of the two key dense granule protein genes, GRA2 and GRA6, which are responsible for shaping the IVN of *T. gondii* [50, 53], possibly because of highly divergent sequences not identifiable by homology [10].

In addition to the digestion of host chromosomes as a putative nutritional resource, intracellular parasites may also rely on the host’s central carbon metabolism for powering their development, replication, and successful production of infectious free-living dinospores. We therefore investigated whether the carbon metabolism and storage capacity of the host were remodeled during infection and identified potential carbon sources that could be utilized for parasite development.

### Impact of parasitic infection on host bioenergetics

**Fig. 3:**
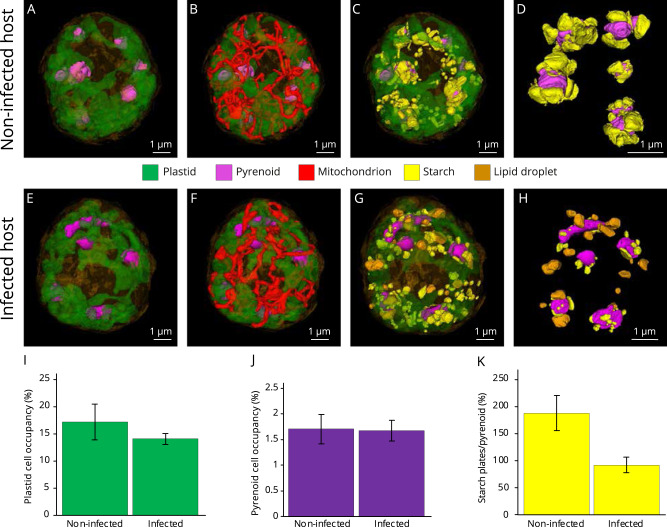
3D cellular architecture of non-infected and infected microalgal host cells (the dinoflagellate *Scrippsiella acuminata*) unveiled by FIB-SEM with a focus on the bioenergetic machinery and carbon reserves. 3D reconstruction of the non-infected host cells with (**A**) its plastid (green) and C-fixing pyrenoids (purple), (**B**) mitochondrion; (**C**) starch grains and plates (yellow); and (**D**) starch plates (yellow) around the pyrenoids (purple). 3D reconstruction of the infected host cells with (**E**) its plastid (green) and pyrenoids (purple), (**F**) mitochondrion; (**G**–**H**) Starch (yellow) and lipids (orange). Volume occupancy (% of the cell volume) of the plastid (**I**) and the pyrenoid (**J**), and the volume ratio between the starch plates and the pyrenoid (K) in three non-infected and three infected hosts cells after FIB-SEM-based 3D reconstruction.

The use of volume electron microscopy allowed for reconstruction and quantification of changes to organelles and subcellular compartments of the algal host that are central for its bioenergetics both before (non-infected hosts) and throughout parasitic infection. We particularly focused on cellular sites for carbon fixation (plastids and pyrenoids) and storage (lipids and starch) in order to assess the impact of the parasite on the central carbon metabolism and carbon partitioning of its host. While the volume occupancy of the mitochondrion tended to be similar between non-infected and infected host cells (4.9% and 4.5% respectively, Table S1), the plastid occupancy only slightly decreased over the course of infection, from 17.2 ± 3.2 % of the cell volume in non-infected cells (*n* = 3) to 14.0 ± 1 % in infected host cells (*n* = 3) (Fig. 3, Table S1). In addition, the ultrastructure of the plastids remained intact with a similar arrangement of thylakoid membranes seen in both non-infected and infected host cells (Fig. S1). Although the number of pyrenoids—a rubisco-containing compartment where CO_2_ is fixed [54]—varied within host cells, the pyrenoid volume occupancy remained stable in the host cell throughout infection (1.7% of the cell volume in both infected and non-infected host cells). The maintenance of both cell volume occupancy and structure of plastids and pyrenoids indicates that the host capability for carbon fixation is not impacted during the first stages of infection, suggesting that sugar production is also likely maintained. These results corroborate a previous study that showed stable quantum yield of photosystem II (F_v_/F_m_) and plastid pigments content, along with continuous expression of plastid-encoded photosynthetic genes during most of the infection [18]. This study also noted that dinospore production was fivefold lower in darkness compared to light conditions [18], highlighting the importance of the host photosynthetic machinery and energy production for successful parasite development and replication. Based on these observations, we apply the term “zombification” to this process, during which the host nucleus is degraded by intranuclear parasites while the host cell remains physiologically active, continuing to swim and maintaining functional energy-producing organelles.

To better evaluate carbon storage in non-infected and infected hosts (cryofixed at the same time), we quantified the volume of starch, which is a semi-crystalline form of storage polysaccharide located in the cytoplasm in the form of grains and attached to the pyrenoid as plates (Fig. 3C–G). The total starch occupancy (grains and plates; reconstructed in 3D) decreased in infected cells, changing from 5% of the cell volume before infection to 3% during infection (Table S1). Starch was even completely absent in one host cell infected by three trophont parasites and was typically almost absent at the final sporulation stage. This overall decrease in starch volume is mainly explained by the two-fold decrease in the volume of the starch plates around the pyrenoid. On average, starch plates were 1.75 times more voluminous than the pyrenoid in non-infected cells and about two times less voluminous than the pyrenoid during infection (Fig. 3D, H, K). Such a decrease in the host sugar reserves suggests two different but potentially simultaneous scenarios: the host machinery for sugar production is (1) functional but is slowed down during the infection, and/or is (2) functional but starch is more rapidly consumed by the host and parasite and/or rewired into the central carbon metabolism of the host. The presence of several host transcripts for cytosolic soluble and granule-bound starch synthases at each infection stage indicates the potential for starch synthesis by the host throughout the infection (Table S4). The host also encodes for plant homologs involved in initial starch mobilization (a cytosolic alpha-glucan and phosphoglucan water dikinase) and enzymes degrading the mobilized starch (beta-amylases and isoamylases) [55], thus potentially providing soluble sugars to the parasite. Transcripts for these host enzymes were found to be present up until the last stage of infection (Table S4). In contrast, we were unable to identify any of these starch degradation genes in the genome of the parasite. It is therefore possible that the parasite scavenges sugar molecules, such as glucose, directly from the host.

**Fig. 4:**
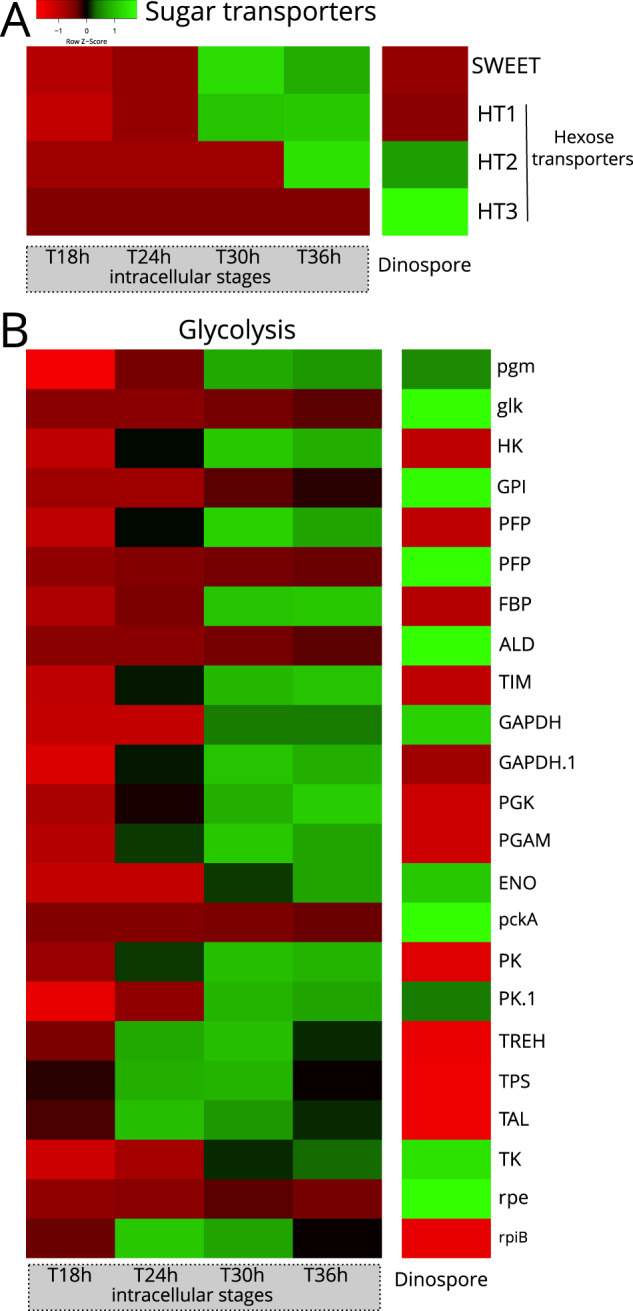
Expression levels of genes involved in sugar transport and glycolysis of the marine parasite *Amoebophrya* across different intracellular stages within its host (the dinoflagellate *Scrippsiella acuminata*) and in dinospores (extracellular). **A** Heatmap showing the expression level of four genes of the parasite encoding putative sugar transporters during the infection (T18 h, T24 h, T30 h and T36 h) and the dinospore stage: one SWEET (Sugars Will Eventually be Exported Transporters) and three hexose transporters (HT1, HT2, and HT3). (See also Figs. S6, S7 and S9, Table S2). **B** Heatmap showing the expression level of genes of the glycolysis pathway of the parasite during the infection (T18 h, T24 h, T30 h and T36 h) and in dinospores. The list of genes, their sequences and expression values can be found in Table S2.

The growth and replication of apicomplexan parasites rely on a continuous supply of host-derived sugars via different transporters [56]. For example, the hexose transporter PfHT1 can transport both glucose and fructose across the cell membrane of *Plasmodium falciparum* [56, 57]. We therefore searched for two families of sugar transporters in the genome of *Amoebophrya* (strain A120): hexose transporters (HT) and Sugars Will Eventually be Exported Transporters (SWEET). Using similarity searches and phylogenetic analyses, we identified one SWEET-like protein predicted to have at least six transmembrane domains (TMs), as well as three hexose transporters (HTs) displaying 11–12 predicted TMs (Figs. S6 and S7). We then investigated their expression level at different stages of the infection and compared them to the dinospore stage. The SWEET gene and one HT (HT1) gene had maximum expression during the intracellular trophont stage (T30 h-T36 h), with nearly no expression in dinospores (Fig. 4A). We hypothesize that SWEET and HT1 are likely involved in sugar scavenging from the host cell during the intracellular development of the parasite. SWEETs are known for bidirectional passive transport of various mono- and disaccharides from high to low sugar concentrations [58, 59]. High concentration of sugars in the host could allow the parasite to “passively” obtain these metabolites through its SWEET transporter without energy consumption. The two other HT genes (HT2 and HT3) were mainly expressed at later intracellular trophont and sporont stages (T36 h) and in dinospores, suggesting a sequential role of these transporters for sugar transport within the cell during the life cycle of the parasite (Fig. 4A).

Lipid droplets are also a major carbon storage site in microalgae. These reserves contain neutral lipids such as cholesterol esters and triacylglycerols (TAG) and have been shown to be key players in host-pathogen interactions [52]. In electron microscopy, lipid droplets are readily recognizable as homogeneous electron-dense structures without a membranous bilayer. Contrary to non-infected host cells, we observed large lipid droplets in the cytoplasm of infected host cells, representing a total volume from 19.4 µm^3^ up to 49 µm^3^ (between 1.33% and 2.3% of the host volume) (Fig. 3G, H). While most lipid droplets were closely associated with the plastid and mitochondrion, some were attached to the host nucleus (Fig. S8). Host transcripts for the complete FASII fatty acid biosynthesis pathway, which provides fatty acids for incorporation into TAGs, were detected throughout infection, as well as several isoforms of diacylglycerol acyltransferase (DGAT; catalyzing the last step of TAG formation), and acetyl-CoA acetyltransferase (ACAT; involved in cholesterol esterification) (Table S4). An increase in host lipid droplets has also been observed during infection by *Toxoplasma* and *Plasmodium* [52, 60, 61] and represents a lipid scavenging strategy [52, 61, 62]. Future investigations to see whether the *Amoebophrya* parasite benefits from this production of lipids in the host will shed light on its metabolic strategy during infection and on evolutionary-conserved strategies in parasitic alveolates across ecosystems and hosts.

Altogether, we provide evidence that the photosynthetic machinery and carbon metabolism of the zombified algal host are still active, but altered, during infection with *Amoebophrya*. The associated production of sugars and lipids could represent an energetic source to fuel the metabolism of the parasite (notably for ATP production). An evaluation of the metabolic activity of the parasite is therefore of primary importance to fully understand the complete life cycle and key adaptations that have evolved to allow the parasite to thrive within an intracellular host environment.

### Metabolism of the intracellular parasite (ATP production)

**Fig. 5:**
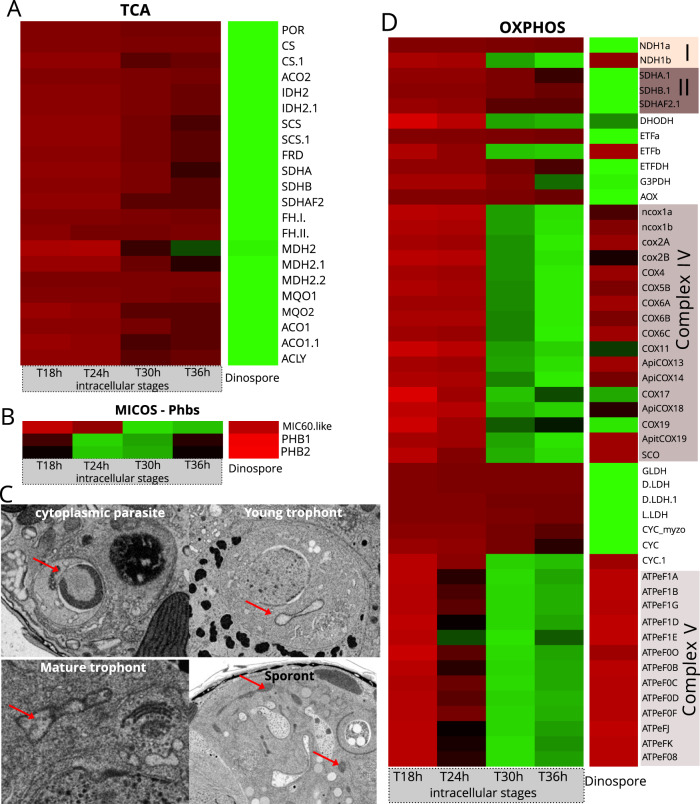
Expression levels of genes involved in mitochondrial respiration and formation of cristae in the parasite *Amoebophrya* across different intracellular stages within its host (the dinoflagellate *Scrippsiella acuminata*) and in dinospores (extracellular). Heatmap showing the expression level of genes of the TCA cycle (**A**) and the OXPHOS (**D**) pathway of the parasite. See also Fig. S10 and Table S2; (**B**) Expression levels of genes encoding MiC60 from the MICOS complex (MItochondrial contact site and Cristae Organizing System), and the prohibitin Phb1 and Phb2 genes. **C** Transmission Electron microscopy (TEM) micrographs showing the internal morphology of the mitochondrion of the parasite at different infection stages. In the cytoplasmic parasite, the electron dense mitochondrion harbored cristae (internal invagination of the inner mitochondrial membrane), which were absent in the mitochondrion of the nuclear trophont parasites (young and mature trophonts). Some vesicles could be observed in the mitochondrion of the mature trophont. Cristae reappeared in the sporont stage where the mitochondrion was substantially developed. The list of genes, their sequences and expression values can be found in Table S2.

**Fig. 6:**
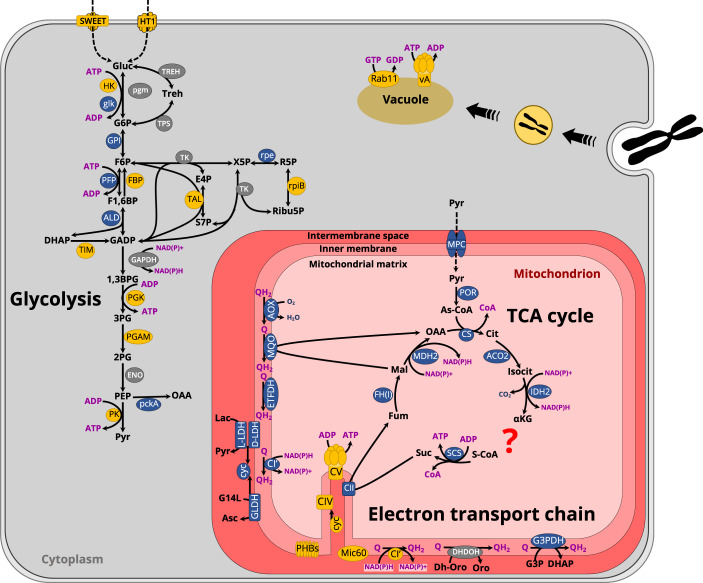
Schematic overview of the potential metabolism of the marine parasite *Amoebophrya* inside its microalgal dinoflagellate host *Scrippsiella acuminata* underlying major metabolic shifts. Major metabolic pathways have been displayed where the color of individual enzymes indicates the stage with maximum expression of their genes: yellow for intracellular parasites; blue in dinospores (free-living stage); gray whereby no difference in expression between the two stages. Dashed lines represent transport of various components; filled lines represent enzymatic reactions. Coenzymes are color-coded as purple: adenosine bi- and triphosphate (ADP and ATP, respectively); nicotinamide adenine dinucleotide phosphate (NAD(P)H); coenzyme A (CoA); quinone pool (Q and QH_2_). The putative location of one of the NADH:ubiquinone oxidoreductase (NDH1b/CI’) complexes, the hexose transporter (HT1) and Sugars Will Eventually be Exported Transporter (SWEET) are depicted by yellow. A question mark in the tricarboxylic acid cycle (TCA) illustrates the apparent loss of the oxoglutarate dehydrogenase/ (OGDC) or α-ketoglutarate dehydrogenase complex in *Amoebophrya*. Invagination of the cytoplasm to form a cytopharynx is represented to illustrate captured host chromosome to be digested in vacuoles. The list of genes, their sequences and expression values can be found in Table S2.

Cellular ATP can be produced by cytoplasmic glycolysis, as well as via the mitochondrial tricarboxylic acid (TCA) cycle and oxidative phosphorylation (OXPHOS) pathways. We reconstructed the glycolysis pathway in *Amoebophrya* and assessed the expression levels of its constituent genes at the intracellular and dinospore stages using time-resolved transcriptomics data (Fig. 4B and S9). Overall, we observed distinct expression patterns where genes of the preparatory phase of glycolysis (consumption of ATP) were expressed in both intracellular and dinospore stages while most genes of the pay-off phase (production of ATP and NAD(P)H) were only expressed during intracellular infection (Fig. 4B). Overall, gene expression analysis suggests that glycolysis of the intracellular parasite is active, which leads to the production of pyruvate that can potentially fuel the TCA cycle. However, we found that all of the genes involved in the TCA cycle were mostly expressed in dinospores (Figs. 5A and 6). Similarly, the Mitochondrial Pyruvate Carrier, that allows pyruvate to enter the mitochondrion was also only expressed in dinospores (Fig. 6). Therefore, these results imply that there is no complete oxidation of carbohydrates and lipids during infection within the host, and the intracellular parasite does not rely on the TCA cycle to produce ATP (and NAD(P)H). This is also the case during the asexual stage of *Plasmodium* that mainly relies on glycolysis to produce ATP, while TCA metabolism occurs at low turnover [63]. This metabolic strategy is commonly found in highly proliferating cells (e.g. apicomplexans, cancer cells) that undergo low respiration and increased glycolysis to support biomass generation with glycolytic intermediates (also known as the Warburg effect) [64]. Such high glycolytic flux typically occurs in glucose-replete environments, which is likely the case here for the parasite *Amoebophrya* when inside the physiologically active host. Reliance on glycolysis and low respiration led to the assumption that the mitochondrion of the parasite *Amoebophrya* might be metabolically quiescent in the intracellular stage. The substantial development of the reticulate mitochondrion during the infection (220-fold increase of the volume) might be unrelated to bioenergetics but rather a mechanism to distribute a mitochondrion among newly forming dinospores (Fig. 1). To further understand mitochondrial activity, we investigated the internal morphology as well as the expression level of genes involved in the OXPHOS pathway. The single electron-dense mitochondrion in cytoplasmic parasites displayed typical cristae (internal invagination of the inner mitochondrial membrane) that are also found in the free-living dinospore [19] (Fig. 5C). Then, in the nuclear young trophont, the mitochondrion developed as an empty “tube” without forming cristae. In the mature trophont, vesicle-like structures were observed in the mitochondrion (Fig. 5C), which could be the initial step of crista biogenesis [65]. Reappearance of canonical cristae only occurred during the sporont stage within the reticulate mitochondrion. This is similar to the development of the mitochondrion of *Plasmodium*, where cristae are also temporarily absent in the asexual blood-stage and reform in the sexual stages (gametocytes) [66–68].

Mitochondrial cristae play a central role in cellular metabolism since they are sites of high concentration of protons (protonic capacitance) where ATP is generated [69]. In eukaryotes, cristae formation and stabilization mainly relies on the interplay of three players, the MItochondrial contact site and Cristae Organizing System (MICOS), the large GTPase optic atrophy 1 (OPA1), and the mitochondrial ATP synthase (F_1_F_0_-ATPase, also known as complex V of OXPHOS) [65, 70, 71]. The presence of cristae is also concomitant with the formation of ring complexes encoded by the prohibitin Phb1 and Phb2 genes [72]. In *Toxoplasma*, the assembly of ATP synthases into hexamers was also shown to be responsible for cristae invagination [73]. By investigating the genome of the parasite *Amoebophrya*, we identified both prohibitin genes but only found one gene (Mic60) of the MICOS complex and no homolog for OPA1. Transcriptomic data revealed similar expression patterns for Mic60 and components of complex V, both displaying maximum expression at T30 h-T36 h and low or no expression in the early infection stages or in dinospores (Fig. 5B, D). Similarly, the prohibitin genes Phb1 and Phb2 were mainly expressed during the intracellular phase with almost no expression in dinospores (Fig. 5B). Thus, gene expression levels suggest that cristae formation and stabilization occur in late intracellular stages and this explains the acristate mitochondrion observed in young trophonts (Fig. 5C).

Cristae formation can be linked to the production of ATP through the dissipation of the proton gradient generated by the mitochondrial Electron Transport Chain (ETC). The OXPHOS pathway in *Amoebophrya* is divided into two independently operating subchains [10], very similar to what has been described in *Chromera velia* [74] (Fig. S10). In *Amoebophrya*, the proton gradient is only generated by the cytochrome C oxidase (complex IV) and is dissipated by complex V [10, 12]. We found that genes of complexes IV and complex V displayed maximum expression at T30 h and T36 h, along with one homolog of cytochrome C (CYC) and NADH:ubiquinone oxidoreductase (NDH1 or CI’) (Figs. 5D and 6, and S10). Such expression patterns suggest that ETC-based ATP production starts to occur during the late intracellular stages of infection, which coincides with the formation of cristae at the sporont stage. These results also indicate that many components of the mitochondrial OXPHOS are dispensable during the intracellular stage, except the Dihydroorotate dehydrogenase (DHODH) for pyrimidine synthesis (Fig. S10), as also seen in the parasite *Plasmodium falciparum* [75].

By contrast, in the dinospore stage, the concomitant expression of all genes of the TCA cycle (including the Mitochondrial Pyruvate Carrier) and most genes of the OXPHOS pathway (excluding complexes IV and V) suggests that the parasite maintains an active catabolism while searching for a new host (Figs. 5 and 6, and S10). Although it is not known whether the parasite can feed *ex hospite*, we hypothesize that the lifespan of the free-living dinospores and the success rate of new infections strongly depend on carbon reserves accumulated from host bioenergetics during the intracellular stages (e.g. sugar, lipids), which can then fuel TCA and OXPHOS in the glucose-deplete oceanic waters. It is worth noting that the free-living dinospores are aerobic [12] and no evidence of fermentative metabolism was found in the transcriptomes of the intracellular stage, which likely experiences permanent oxygenated environment due to host photosynthesis.

## Conclusion

By combining transcriptomics and 3D electron microscopy, we shed new light on intracellular development of the marine parasite *Amoebophrya* within its microalgal host, and unveil dramatic shifts in trophic strategy and metabolic activity. Upon entry into the host, the cytoplasmic parasite appears to be metabolically and transcriptionally quiescent until it rapidly invades the host nucleus. There, the growing trophont undergoes major morphological changes, particularly following the trophic switch into phagotrophy, when host chromosomes are engulfed and digested. We hypothesize that this “zombification” of the host cell and trophic strategy switch provides a highly valuable source of carbon and nutrients for the development and growth of the parasite. This ability to invade the host nucleus is not unique among parasites as this process has been described in some coccidians (e.g. *Eimeria*) that cause intranuclear coccidiosis in vertebrates [76, 77].

Additionally, intracellular parasites likely benefit from host sugar production to fuel glycolysis for ATP production. Since the zombified host remains photosynthetically active with preserved plastids and pyrenoids, production of sugar is likely maintained throughout infection. It is also possible that a lipid scavenging strategy enables the parasite to benefit from host lipid droplets, which were observed to increase during infection. ATP production in the intracellular parasite can also occur at late stages through the activity of mitochondrial complexes IV and V. This is reflected in the morphological plasticity of the parasite mitochondrion, which first expands without cristae in the nuclear trophont parasites. Mitochondrial cristae then reappear at the sporont stage before the expanded mitochondrion is distributed among individual dinospores, which require higher levels of respiration. Indeed, we provide evidence that the free-living dinospore stage of the parasite switches energy metabolism towards oxidative phosphorylation and may rely on carbon reserves salvaged and accumulated during intracellular development.

It is interesting to note that the intracellular development and metabolism of the marine planktonic parasite *Amoebophrya* exhibits many similarities with the apicomplexans *Toxoplasma gondii* and *Plasmodium falciparum*, as well as the kinetoplastid *Trypanosoma* brucei. These parasites also have highly flexible mitochondrial development and most of them (except *T. brucei*) display a sequential acristate and cristate mitochondrion during their intracellular life cycle [37, 68, 78, 79]. This mitochondrial plasticity can be linked to their metabolic strategy with high glycolytic activity and low respiration at one particular stage of infection, but also to the cell cycle of the parasite where a mitochondrion must be distributed to each daughter cell [78]. Like in apicomplexans, we also observed the IVN-like network, synthesis of lipid droplets in the host upon infection, and phagotrophic activity to ingest host material [52, 53, 80]. These mechanisms appear to be evolutionarily conserved or metabolically constrained across parasitic alveolates in different ecosystems regardless of their eukaryotic hosts, thus underlining their importance during infection.

Future investigations are now required to study the role of the IVN-like network, as well as the molecular players that are involved in phagotrophy and sugar and lipid scavenging in this marine parasite. Metabolomics will also have the potential to improve our understanding of the metabolic rewiring of the parasite during infection and identify potential metabolites scavenged from the host. This knowledge will be essential to elucidate the survival of the free-living dinospores and understand the ecological success of this widespread parasite, which has a major impact on phytoplankton populations and therefore carbon cycling in the ocean.

## Supplementary information

supplementary method

supplementary figures

Table S1

Table S2

Table S3

Table S4

## Data Availability

Raw electron microscopy data are deposited in EMPIAR, accession code EMPIAR- 47484134. The genomic data (DNA and RNA-seq data) can be found on EMBL-EBI BioProject PRJEB39972 and assemblies are available at http://application.sb-roscoff.fr/blast/hapar/download.html.
