# Supplementary material for: Intracellular development and impact of a marine eukaryotic parasite on its zombified microalgal host
Source: ISME J. 2022 Jul 8;16(10):2348–59. doi: 10.1038/s41396-022-01274-z (PMC9478091; doi:10.1038/s41396-022-01274-z)
Supplement: Supplementary file 1 — supplementary method [file 41396_2022_1274_MOESM1_ESM.docx]

**Identification of host transcripts:**

Known proteins (starch synthases, proteins of the FASII fatty acid biosynthesis pathway) of the dinoflagellate *Scrippsiella trochoidea*, closely related to the host (*Scrippsiella acuminata*), were used as queries against the un-infected host transcriptome. The respective best hit (or >1 hit, if several isoforms of one transcript were identified) were added to existing phylogenies for the corresponding protein ^1,2^ to confirm the identity of the retrieved host transcripts. For the proteins involved in starch degradation, acetyl-CoA carboxylase (ACC) and the proteins involved in lipid droplet biosynthesis, new phylogenies were generated, using annotated proteins from *Arabidopsis thaliana* and/or *Homo sapiens* as queries (downloaded from <https://www.uniprot.org>; last access October 2021). The queries were used in a BLASTP search against a comprehensive custom database containing representatives from all major eukaryotic groups (except excavates) and RefSeq data from all bacterial phyla at NCBI (last accessed December 2017). The database was subjected to CD-HIT (https://pubmed.ncbi.nlm.nih.gov/23060610/) with a similarity threshold of 85% to reduce redundant sequences and paralogs. The search results of the BLASTP step were parsed for hits with an e-value threshold ≤1e-25 and a query coverage of ≥ 50% to reduce the possibility of paralogs and short sequences. The number of bacterial hits was restrained to 20 hits per phylum (for FCB group, most classes of Proteobacteria, PVC group, Spirochaetes, Actinobacteria, Cyanobacteria (unranked) and Firmicutes) or 10 per phylum (remaining bacterial phyla) as defined by NCBI taxonomy. Parsed hits were aligned with MAFFT v. 7.480, using the --auto option, poorly aligned regions were eliminated using trimAl v. 1.2 (https://pubmed.ncbi.nlm.nih.gov/19505945/) with a gap threshold of 80% and Maximum likelihood tree reconstructions were performed with FastTree v. 2.1.7 using the default options. The resulting phylogenies were inspected in FigTree v1.4.4, and recovered *S. trochoidea* hits were, as described above, used to identify homologs in the host transcriptome. Tree reconstruction and visual inspection were repeated including the host candidates. Complex, unresolved phylogenies (DGAT, ACAT) were further investigated by first manually inspecting the initial phylogenies and underlying alignments to remove contaminating, divergent and/or low-quality sequences. The cleaned, unaligned sequences were then subjected to filtering with PREQUAL using the default options (https://pubmed.ncbi.nlm.nih.gov/29868763/) to remove non homologous residues introduced by poor-quality sequences, followed by alignment with MAFFT G-INS-i using the VSM option (--unalignlevel 0.6) to control over-alignment ^3^. The alignments were subjected to Divvier ^4^ using the -divvygap and the -mincol 4 option to improve homology inference before removing ambiguously aligned sites with trimAl (-gt 0.01). Final trees were calculated with IQ-TREE v. 1.6.5 ^5^, using the -mset option to restrict model selection to LG for ModelFinder ^6^, while branch support was assessed with 1000 ultrafast bootstrap replicates ^7^. In addition, all putative DGAT and ACAT candidates in the host were submitted to InterProScan at <https://www.ebi.ac.uk/interpro/> to confirm their identity ^8^.

1. Hehenberger, E., Burki, F., Kolisko, M. & Keeling, P. J. Functional Relationship between a Dinoflagellate Host and Its Diatom Endosymbiont. *Mol. Biol. Evol.* **33**, 2376–2390 (2016).

2. Hehenberger, E., Gast, R. J. & Keeling, P. J. A kleptoplastidic dinoflagellate and the tipping point between transient and fully integrated plastid endosymbiosis. *Proc. Natl. Acad. Sci. U. S. A.* **116**, 17934–17942 (2019).

3. Katoh, K., Rozewicki, J. & Yamada, K. D. MAFFT online service: multiple sequence alignment, interactive sequence choice and visualization. *Brief. Bioinform.* **20**, 1160–1166 (2019).

4. Ali, R. H., Bogusz, M., Whelan, S. & Tamura, K. Identifying Clusters of High Confidence Homologies in Multiple Sequence Alignments. *Mol. Biol. Evol.* **36**, 2340–2351 (2019).

5. Nguyen, L. T., Schmidt, H. A., Von Haeseler, A. & Minh, B. Q. IQ-TREE: A fast and effective stochastic algorithm for estimating maximum-likelihood phylogenies. *Mol. Biol. Evol.* **32**, 268–274 (2015).

6. Kalyaanamoorthy, S., Minh, B. Q., Wong, T. K. F., von Haeseler, A. & Jermiin, L. S. ModelFinder: fast model selection for accurate phylogenetic estimates. *Nat. Methods* **14**, 587–589 (2017).

7. Hoang, D. T., Chernomor, O., von Haeseler, A., Minh, B. Q. & Vinh, L. S. UFBoot2: Improving the Ultrafast Bootstrap Approximation. Molecular biology and evolution. *Mol. Biol. Evol.* **35**, 518–522 (2018).

8. Blum, M. *et al.* The InterPro protein families and domains database: 20 years on. *Nucleic Acids Res.* **49**, D344–D354 (2021).
